# Late‐Onset Tay–Sachs Disease With SMALED‐Like Muscle MRI Pattern Despite a Distinct Clinical Phenotype

**DOI:** 10.1111/jns.70144

**Published:** 2026-07-17

**Authors:** Rodrigo Siqueira Soares Frezatti, Trajano Aguiar Pires Gonçalves, Manoella Guerra de Albuquerque Bueno, André Clériston José dos Santos, Lindsay A. Wilson, Natalia Dominik, Michael G. Hanna, Jasper Morrow, Alex M. Rossor, Mary M. Reilly, Pedro José Tomaselli, Wilson Marques

**Affiliations:** ^1^ Department of Neurosciences and Behaviour Sciences, Neuromuscular Disorders University of São Paulo Ribeirao Preto Brazil; ^2^ Department of Neuromuscular Diseases, Centre for Neuromuscular Diseases UCL Queen Square Institute of Neurology London UK; ^3^ NHS Highly Specialised Service for Rare Mitochondrial Disorders Queen Square Centre for Neuromuscular Diseases, The National Hospital for Neurology and Neurosurgery London UK; ^4^ Instituto Nacional de Ciência e Tecnologia Translacional em Saúde Mental Digital (INCT‐SMD) Porto Alegre Rio Grande do Sul Brazil

**Keywords:** HEXA, late‐onset Tay–Sachs disease, muscle MRI, non‐5q spinal muscular atrophy, phenocopy, spinal muscular atrophy with lower extremity predominance

## Abstract

**Background:**

Late‐onset Tay–Sachs disease (LOTS) is a rare lysosomal disorder that contrasts with the classical infantile form by presenting with milder and heterogeneous neurological manifestations, including lower motor neuron phenotypes. While muscle MRI fatty infiltration patterns have been described in selected inherited motor neuron disorders, the corresponding imaging features in LOTS remain poorly defined.

**Case:**

We report two female patients presenting in the second decade of life with slowly progressive, lower limb–predominant flaccid weakness associated with tremor. Hip flexion and arm extension were disproportionately affected. Electromyography demonstrated a neurogenic pattern. Whole‐body muscle MRI (wbMRI) revealed selective fatty infiltration on T1‐weighted sequences with normal STIR imaging. At the pelvic and thigh levels, this pattern showed marked overlap with the characteristic fatty infiltration described in SMA with lower extremity predominance (SMALED), including relative sparing of the adductor longus and semimembranosus. In contrast, consistent additional involvement of the iliopsoas and triceps brachii muscles was observed. Whole‐exome sequencing identified compound heterozygous pathogenic variants in the HEXA gene in each patient, confirmed to be in trans, and enzymatic testing demonstrated reduced β‐hexosaminidase A activity.

**Conclusion:**

LOTS may reproduce key elements of the SMALED muscle MRI fatty infiltration pattern, particularly at the pelvic and thigh levels, while maintaining a distinct clinical presentation. Recognition of this partial radiological overlap, together with its discriminative features, is important to avoid diagnostic misclassification and to guide appropriate genetic testing.

## 1 Introduction

Tay–Sachs disease (TSD) is a rare autosomal recessive lysosomal storage disorder caused by deficiency of the α‐subunit of β‐hexosaminidase A (HexA), a heterodimeric enzyme composed of α and β subunits responsible for the degradation of GM2 gangliosides within lysosomes [1]. Pathogenic variants in *HEXA* impair GM2 catabolism, leading to progressive neuronal accumulation and neurodegeneration [1, 2].

The clinical spectrum of TSD is largely determined by residual HexA activity. Severe enzyme deficiency, most often associated with null *HEXA* variants, causes the classic infantile form, characterized by early‐onset developmental regression, seizures, pyramidal signs, and rapid neurological deterioration, typically resulting in death in early childhood [1, 2]. In contrast, hypomorphic *HEXA* variants, frequently occurring in compound heterozygosity, allow partial enzymatic activity and give rise to later‐onset phenotypes with marked clinical heterogeneity [3]. In parallel, advances in diagnosis and increasing development of disease‐modifying strategies for GM2 gangliosidoses, including substrate reduction and gene‐based therapies currently under clinical investigation, have emphasized the importance of early and accurate identification of affected individuals [3, 4].

Late‐onset Tay–Sachs disease (LOTS) may present in adolescence or adulthood with a broad range of neurological manifestations, including cerebellar ataxia, psychiatric symptoms, dystonia, peripheral neuropathy, or neuromuscular syndromes, often complicating and delaying diagnosis [4, 5]. Among these, LOTS has been repeatedly reported in the differential diagnosis of amyotrophic lateral sclerosis and non‐5q spinal muscular atrophies (SMA) [3, 5, 6].

In presentations dominated by lower motor neuron involvement, patients typically exhibit slowly progressive weakness, reduced or absent reflexes, with neurogenic changes on electromyography, and absence of sensory involvement [3]. Classical clinical features, such as cerebellar ataxia, extrapyramidal signs, or cherry‐red macula, are frequently absent in this context. Nevertheless, specific patterns of weakness have been noted. Riboldi et al. described a characteristic predominance of hip flexion weakness in the lower limbs and disproportionate arm extension weakness in the upper limbs, referred to as the “triceps sign” [7]. Lower limb–predominant neurogenic weakness has also been described in LOTS, sometimes raising diagnostic consideration of non‐5q SMA [3, 8]. To date, imaging descriptions in LOTS are restricted to isolated cases or small series [3], lacking a systematic whole‐body muscle MRI (wbMRI) characterization.

In this report, we describe two unrelated patients with LOTS presenting with a lower motor neuron syndrome characterized by lower limb predominance and disproportionate involvement of hip flexion and arm extension. wbMRI demonstrated selective fatty infiltration, with a pattern at the pelvic and thigh levels showing marked overlap with that described in SMA with lower extremity predominance (SMALED). However, consistent additional involvement of the iliopsoas and triceps muscles was observed, representing a distribution not previously described in SMALED.

## 2 Case Report

Case 1 was a 34‐year‐old woman born to nonconsanguineous parents, with normal birth, neurodevelopment, academic performance, and preserved cognition. At 13 years of age, she developed a symmetrical upper limb rest tremor. Over subsequent years, she experienced slowly progressive weakness predominantly affecting the lower limbs, with clear proximal predominance. From symptom onset, she reported disproportionate difficulty with activities requiring hip flexion, including climbing stairs, rising from a seated position, and getting into a car. Upper limb involvement emerged later, predominantly affecting arm extension and hand dexterity.

Neurological examination at 25 years of age showed a waddling gait and Gowers' sign. Using the 0–5 Medical Research Council (MRC) scale demonstrated symmetric, nonlength‐dependent weakness: neck flexion 5, neck extension 5; arm abduction 3, arm adduction 4; elbow flexion 4+, elbow extension 2–3; wrist flexion 4, wrist extension 4+; finger extension 4, intrinsic hand muscles 4; hip flexion 2, hip extension 3; leg adduction 4+, leg abduction 4+; knee extension 3, knee flexion 4−; ankle dorsiflexion 2, ankle plantar flexion 2; toe extension 2, and toe flexion 2. Deep tendon reflexes were preserved in the upper limbs and absent in the lower limbs. A mild rest tremor was present without bradykinesia or rigidity. Sensory examination, cranial nerve examination, cognition, and fundoscopy were normal, with no cherry‐red macula, psychiatric or extrapyramidal features.

Nerve conduction studies demonstrated normal sensory nerve action potentials and reduced compound muscle action potential amplitudes in the lower limbs. Electromyography in both upper and lower limbs showed diffuse chronic denervation with a proximal and non–length‐dependent distribution. MLPA analysis revealed two copies of SMN1.

Case 2 was a 32‐year‐old woman born to nonconsanguineous parents, with normal birth, neurodevelopment, and preserved cognitive function. At 15 years of age, she developed a mild, symmetrical, bilateral resting tremor affecting the upper limbs. In subsequent years, she experienced slowly progressive, predominantly proximal weakness, more marked in the lower limbs. Similar to Case 1, she reported difficulties performing activities dependent on hip flexion. In early adulthood, she developed frequent falls, becoming wheelchair dependent for long distances by 21 years of age. Upper limb weakness emerged later, at age 25, predominantly affecting arm extension.

Neurological examination at 19 years of age revealed a highly similar pattern of non–length‐dependent weakness, as shown in power examination graded by the 0–5 MRC scale: neck flexion 5, neck extension 5; arm abduction 3, arm adduction 4; elbow flexion 4+, elbow extension 2–3; wrist flexion 4, wrist extension 4+; finger extension 4, intrinsic hand muscles 4; hip flexion 2, hip extension 3; leg adduction 4+, leg abduction 4+; knee extension 3, knee flexion 4−; ankle dorsiflexion 2, ankle plantar flexion 2; toe extension 2, and toe flexion 2. Deep tendon reflexes, sensory, cranial nerve, cognitive, and extrapyramidal examinations were normal.

Whole‐exome sequencing (WES) in Case 1 identified two heterozygous HEXA variants (NM_000520.6): c.533G>A (p.Arg178His) and c.782G>A (p.Gly261Asp). The p.Arg178His variant is classified as pathogenic in ClinVar and has been repeatedly reported in TSD, including attenuated phenotypes, both in homozygosity and compound heterozygosity, consistently associated with late‐onset or subacute forms rather than infantile disease [9, 10]. The p.Gly261Asp variant affects a conserved residue, is predicted deleterious by in silico tools, and is absent in homozygosity in gnomAD v4. Sanger sequencing confirmed segregation in trans, supporting compound heterozygosity.

β‐Hexosaminidase analysis demonstrated mildly reduced absolute HexA activity (500.0 nmol/h/mL; reference 550–1675), reduced total hexosaminidase activity, and preserved relative HexA fraction (62%), consistent with partial HexA deficiency.

In Case 2, WES identified two heterozygous HEXA variants: c.1495C>T (p.Arg499Cys) and c.805G>A (p.Gly269Ser), both classified as pathogenic in ClinVar and affecting conserved residues. β‐Hexosaminidase analysis showed markedly reduced absolute HexA activity (129 nmol/h/mL; reference 550–1675), reduced total hexosaminidase activity, and a reduced relative HexA fraction (38%), confirming partial HexA deficiency.

**FIGURE 1:**
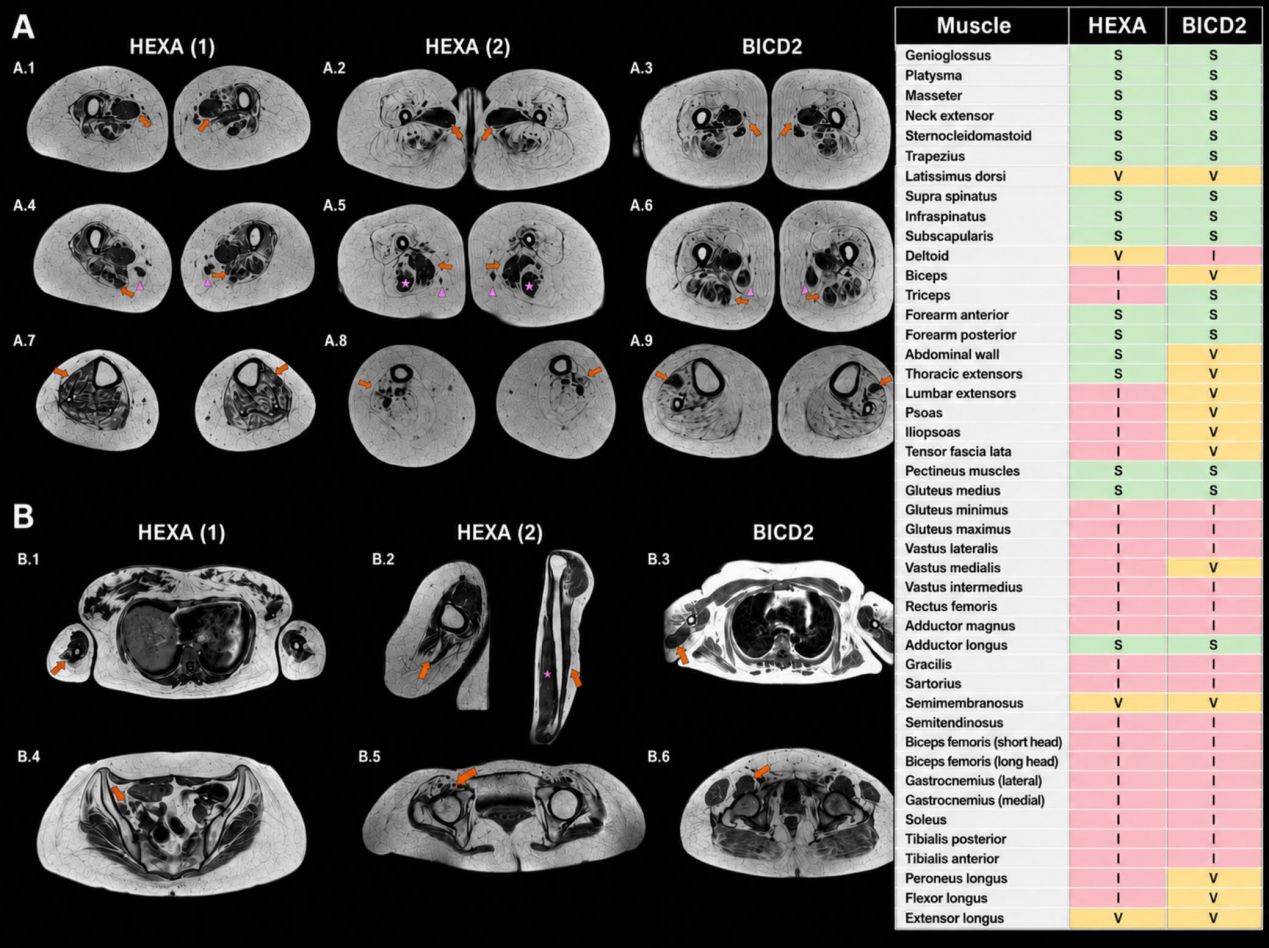
Composite muscle magnetic resonance imaging in late‐onset Tay–Sachs disease and BICD2‐related spinal muscular atrophy with lower extremity predominance. (A.1)–(A.9) Axial T1‐weighted thigh and leg muscle MRI from patients with late‐onset Tay–Sachs disease (HEXA1 and HEXA2) compared with a patient with BICD2‐related spinal muscular atrophy with lower extremity predominance (SMALED). (A.1) and (A.2) the proximal thigh level in HEXA1 and HEXA2, respectively, and (A.3) the corresponding level in BICD2‐related SMALED. (A.4) and (A.5) the distal thigh level in HEXA1 and HEXA2, respectively, and (A.6) the corresponding level in BICD2‐related SMALED. In the LOTS patients, the vasti muscles, semimembranosus, biceps femoris, and sartorius are involved, whereas the adductor muscles (orange arrows), gracilis (magenta arrowheads), and semitendinosus (magenta stars) are relatively spared. (A.7) and (A.8) the leg level in HEXA1 and HEXA2, respectively, and (A.9) the corresponding level in BICD2‐related SMALED. In the legs, despite different disease severity, relative sparing of toe extensors (orange arrows) can be appreciated. The table inset summarizes qualitative T1 assessment: S = spared, I = invariably involved, and V = variably involved. (B.1)–(B.6) T1‐weighted arm and pelvic muscle MRI from the same LOTS patients compared with a patient with BICD2‐related SMALED. (B.1) Arm and upper trunk imaging in HEXA1. (B.2) Arm imaging in HEXA2, including relative sparing of the biceps (magenta star) with triceps involvement (orange arrow). (B.3) The corresponding upper trunk and arm level in the BICD2 comparator, in whom triceps brachii is spared. (B.4) and (B.5) the pelvic level in HEXA1 and HEXA2, respectively, with iliopsoas fatty infiltration (orange arrows), whereas (B.6) the corresponding pelvic level in the BICD2 comparator, in whom the iliopsoas is spared. These findings highlight possible MRI clues that may help differentiate HEXA‐related disease from traditional SMALED genes.

wbMRI in both patients demonstrated a symmetric pattern of selective fatty infiltration on T1‐weighted sequences with normal STIR imaging, indicating a chronic, noninflammatory process. Fatty infiltration predominantly affected the triceps brachii in the upper limbs; the iliopsoas, gluteus medius, and gluteus minimus at the pelvic level; and anterior and posterior compartments of the thighs and legs, with relative sparing of the adductor longus, semitendinosus, and, to a lesser extent, toe extensors (Figure 1).

Whole‐body MRI patterns from both LOTS patients were systematically compared with those from two age‐ and sex‐matched patients from our cohort with genetically confirmed BICD2‐related SMALED, used as a reference for the canonical SMALED imaging pattern. A structured qualitative assessment based on the Mercuri 5‐point fatty infiltration scale was applied. Muscles were classified as spared (≤ 1), involved (≥ 2), or variable if discordant across patients. This comparative approach demonstrated a marked overlap between LOTS and SMALED at the pelvic and thigh levels, with consistent additional involvement of the iliopsoas and triceps muscles in LOTS (Figure 1 and Table 1).

**TABLE 1:** MRC‐based muscle strength assessment and corresponding qualitative muscle MRI status in the two LOTS patients.

Movement tested	Case 1 MRC	Case 2 MRC	Main contributing muscles considered	MRI status (HEXA/LOTS)
Neck flexion	5	5	Sternocleidomastoid/scalene muscles	S
Neck extension	5	5	Neck extensors	S
Arm abduction	3	3	Deltoid and supraspinatus	V
Arm adduction	4−	4	Latissimus dorsi, pectoral and subscapularis muscles	V
Elbow flexion	4+	4+	Biceps brachii/brachialis	S
Elbow extension	2–3	3	Triceps brachii	I
Wrist flexion	4	4	Anterior forearm compartment	S
Wrist extension	4+	4+	Posterior forearm compartment	S
Finger extension	4	4	Posterior forearm/extensor compartment	S
Intrinsic hand muscles	4−	4	Intrinsic hand muscles	Not directly scored
Hip flexion	2	2	Iliopsoas	I
Hip extension	3	4−	Gluteus maximus and hamstrings	V
Leg adduction	4+	4	Adductor longus, adductor magnus and pectineus	V
Leg abduction	4−	4+	Gluteus medius/minimus	I
Knee extension	3	3	Quadriceps femoris	I
Knee flexion	4−	4	Hamstrings	V
Ankle dorsiflexion	2	2	Tibialis anterior and toe extensors	V
Ankle plantar flexion	2	2	Gastrocnemius, soleus and long toe flexors	I
Toe extension	2	2	Long toe extensors	V
Toe flexion	2	2	Long toe flexors	I

*Note:* MRI status follows the qualitative T1‐weighted whole‐body MRI code used in Figure 1: S = spared, I = invariably involved, and V = variably involved across the two HEXA/LOTS patients. For movements with more than one major contributing muscle, the MRI status reflects the predominant or mixed pattern of the corresponding muscle group. Intrinsic hand muscles were clinically tested but were not directly scored in the qualitative whole‐body MRI table.

Abbreviations: LOTS = Late‐onset Tay–Sachs disease; MRC = Medical Research Council; MRI = magnetic resonance imaging.

## 3 Discussion

Spinal muscular atrophy with lower extremity predominance (SMALED) represents a subgroup of non‐5q SMA classically associated with pathogenic variants in DYNC1H1 (SMALED1) and BICD2 (SMALED2) [11, 12]. Although genetically distinct, these conditions converge mechanistically on impaired axonal motor transport, either through direct disruption of the dynein motor complex or through altered cargo recruitment. This shared pathogenic pathway is also reflected in overlapping imaging features, with lower limb–predominant involvement and a characteristic pattern of muscle fatty infiltration on MRI, particularly at the pelvic and thigh levels, with predominant involvement of the gluteus minimus and medius, vasti muscles, semimembranosus, biceps femoris long head, and sartorius, alongside relative sparing of the adductor muscles, semitendinosus, and gracilis. In the legs, diffuse fatty infiltration with relative preservation of toe extensors and peroneal muscles has been consistently reported [13, 14, 15, 16]. This imaging signature is widely used to guide targeted genetic testing and to support variant interpretation in DYNC1H1 and BICD2. By contrast, consistent involvement of specific upper limb muscles has not been systematically described in SMALED.

Although LOTS is a recognized cause of lower motor neuron syndromes and has been reported in the differential diagnosis of amyotrophic lateral sclerosis and adult‐onset non 5q SMA, its muscle MRI characteristics remain poorly characterized and are rarely incorporated into diagnostic algorithms [3, 5]. In both of our patients, wbMRI demonstrated a pattern showing overlap with the canonical SMALED radiological signature, particularly at the pelvic and thigh levels. However, this overlap was incomplete and accompanied by consistent additional involvement of the triceps brachii in the upper limbs and the iliopsoas at the pelvic level, a distribution not described in BICD2‐ or DYNC1H1‐related SMALED. The use of BICD2‐related SMALED as the imaging comparator reflects the availability of muscle MRI data from our cohort. DYNC1H1‐related SMALED was not directly included because comparable images were unavailable, but the available literature supports a highly overlapping canonical SMALED radiophenotype across BICD2‐ and DYNC1H1‐related disease [16]. Therefore, the absence of DYNC1H1 images should be considered a limitation of the visual comparison rather than evidence against including DYNC1H1‐related SMALED in the differential diagnosis.

While the lower limb pattern may resemble SMALED, the clinical phenotype in both patients is clearly divergent. This reinforces that muscle MRI, although highly informative, should not override clinical characterization in syndrome definition. Rather, the integration of clinical and imaging features provides a more reliable framework for differential diagnosis.

These observations also align with previously reported clinical features in LOTS, including preferential involvement of hip flexion and arm extension [7], and extend them to the radiological domain. Together with earlier reports of LOTS presenting with lower motor neuron syndromes ([3, 8]), our findings indicate that HEXA‐related disease can reproduce key elements of the SMALED muscle MRI pattern while maintaining a distinct clinical identity. Recognition of this partial radiological overlap, alongside its discriminative features, particularly upper limb involvement and selective iliopsoas and triceps fatty infiltration, may help refine the diagnostic evaluation of patients with suspected LOTS.

## 4 Conclusion

This report highlights LOTS as an important differential diagnosis in patients presenting with lower motor neuron syndromes, particularly when muscle MRI demonstrates a pattern overlapping with that described in SMALED at the pelvic and thigh levels, alongside prominent involvement of the iliopsoas and triceps brachii muscles. Recognition of this presentation is clinically relevant for accurate diagnosis, genetic counseling, and appropriate referral in the context of emerging disease‐modifying therapies for GM2 gangliosidoses.

## Funding

P.J.T. and R.S.S.F. were supported by an Medical Research Council strategic award to establish an International Centre for Genomic Medicine in Neuromuscular Diseases (ICGNMD) MR/S005021/1. W.M.Jr. is funded by the National Council for Scientific and Technological Development (CNPq) and the National Institute of Science and Technology in Digital Mental Health (INCT–SMD), Grant No. 409148/2024‐5 [Sistema Integrado de Protocolo e Arquivo do Ministério da Saúde: 25000.160.096/2014‐07]; Bolsa de Produtividade em pesquisa. Chamada CNPq Nº 18/2024. Processo: 312821/2025‐5 Conselho Nacional de Pesquisa (CNPq) [310378/2021‐4].

## Ethics Statement

This case was approved by the local ethics committee.

## Consent

The patient provided written informed consent for the use of her clinical data.

## Conflicts of Interest

The authors declare no conflicts of interest.

## Data Availability

Data sharing not applicable to this article as no datasets were generated or analysed during the current study.
